# EGCG alleviates vascular calcification via the MAPK/JunB signaling pathway

**DOI:** 10.1016/j.gendis.2024.101237

**Published:** 2024-02-01

**Authors:** Tiantian Li, Fei Fang, Hongmei Yin, Zhen Zhang, Xiangxiu Wang, Erxiang Wang, Hongchi Yu, Yang Shen, Guixue Wang, Weihong He, Xiaoheng Liu

**Affiliations:** a Institute of Biomedical Engineering, West China School of Basic Medical Sciences & Forensic Medicine, Sichuan University, Chengdu, Sichuan 610041, China; b West China School of Pharmacy, Sichuan University, Chengdu, Sichuan 610041, China; c Department of Cardiology, The Third People's Hospital of Chengdu, Affiliated Hospital of Southwest Jiaotong University, Chengdu, Sichuan 610000, China; d Key Laboratory for Biorheological Science and Technology of Ministry of Education, State and Local Joint Engineering Laboratory for Vascular Implants, Bioengineering College of Chongqing University, Chongqing 400030, China; e JinFeng Laboratory, Chongqing 401329, China; f Department of Physiology, West China School of Basic Medical Sciences & Forensic Medicine, Sichuan University, Chengdu, Sichuan 610041, China

Vascular calcification is a pathophysiological change characterized by abnormal deposition of hydroxyapatite crystals in the vascular walls, contributing to increased morbidity and mortality from cardiovascular diseases.^1^ Currently, there are no effective therapeutic strategies to prevent or impede the progression of vascular calcification. Recent studies have elucidated that inhibiting the phenotypic transition of vascular smooth muscle cells (VSMCs) from contractile to osteo/chondrogenic phenotype represents a pivotal strategy for restraining vascular calcification.^2^ Diet and lifestyle are strongly linked to arterial calcification.^3^ Green tea is the second most popular beverage globally besides water. Studies have substantiated the favorable impact of tea beverages on cardiovascular ailments.^4^ However, the potential of green tea in the investigation of treating and preventing vascular calcification has not been fully explored yet. In this study, we have discovered that (-)-epigallocatechin gallate (EGCG), a prominent catechin present in green tea, exhibits the potential to mitigate vascular calcification both *in vivo* and *in vitro.* Mechanistically, EGCG effectively mitigates vascular calcification by inhibiting mineral deposition and osteogenic differentiation of VSMCs through the MAPK-JunB signaling axis.

Our study used a subcutaneous injection of vitamin D3 to establish the media arterial calcification mouse model. Alizarin Red S staining of entire aortas showed that vitamin D3 induced aortic calcification in the model mice. After 8 weeks of intragastric EGCG administration, the calcium salt deposition and calcium contents in the aorta of calcified mice were significantly reduced (Fig. 1A; Fig. S1A). Alizarin Red S and von Kossa staining of vitamin D3-treated mouse aorta sections also indicated that EGCG treatment significantly decreased calcium deposition (Fig. 1B). In addition, the vascular structure of aortas in the model group was ruptured, combined with a loss of normal wavy contractions, whereas, after EGCG treatment, the elastic fibers of the vessels were continuous and regular (Fig. 1B). *In vitro*, a high phosphate and calcium-induced VSMCs model was used to investigate the effect of EGCG on VSMCs calcification. Alizarin Red S staining confirmed that EGCG reduced calcium deposition of human aortic smooth muscle cells (HASMCs) in a concentration-dependent manner (Fig. 1C). The above findings suggest that EGCG effectively inhibits vascular calcium salt deposition in both animal and cell models.

**Figure 1:**
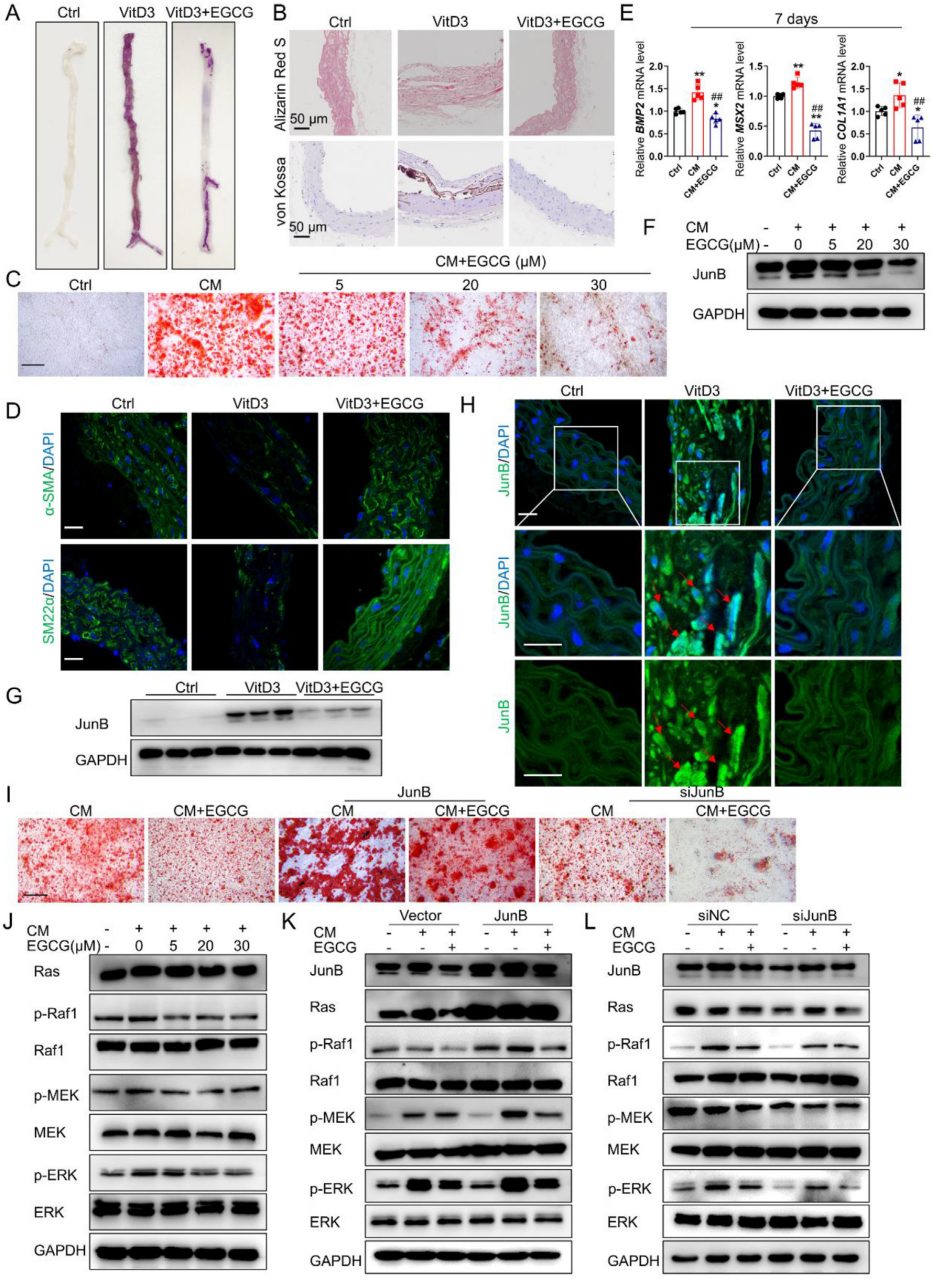
EGCG ameliorated vascular calcification via the MAPK/JunB signaling pathway. **(A)** Representative image of Alizarin Red S staining of aortas. The calcium deposition of the entire aortas was determined by Alizarin Red S staining. The quantification results are shown in Fig. S6. **(B)** Representative Alizarin Red S and von Kossa staining of mouse aorta sections. The quantification results are shown in Fig. S6. **(C)** HASMCs were cultured in growth medium (Ctrl), calcifying medium (CM), or CM with EGCG (5, 20, and 30 μM) for 21 days. Calcium deposition in HASMCs was detected by Alizarin red S staining. Scale bar = 100 μm. **(D)** The expression of α-SMA and SM22α in mouse aortas of indicated groups was analyzed with immunofluorescence assays. Scale bar = 20 μm. The quantification results are shown in Fig. S7. **(E)** HASMCs were cultured in Ctrl, CM, or CM with EGCG (20 μM) for 7 days. mRNA expression of *BMP2*, *MSX2*, and *COL1A1* in HASMCs was detected with quantitative real-time PCR. The data are shown as mean ± standard error of the mean. ∗*P* < 0.05, ∗∗*P* < 0.01 *vs.* Ctrl; ^#^*P* < 0.05, ^##^*P* < 0.01 *vs.* CM (*n* = 5). **(F, G)** The protein expression of JunB in HASMCs and mouse aortas was detected with western blotting. **∗**The quantification results are shown in Fig. S8. **(H)** The expression of JunB in mouse aortas was analyzed with immunofluorescence assays. Scale bar = 20 μm. The quantification results are shown in Fig. S7. **(I)** HASMCs transfected with plasmid and siRNA targeting JunB were cultured in CM or CM with EGCG (20 μM) for 21 days. Calcium deposition in HASMCs was detected by Alizarin Red S staining. Scale bar = 100 μm. **(J)** MAPK pathway-related protein expression in HASMCs of indicated groups was detected with western blotting. The quantification results are shown in Fig. S8. **(K, L)** HASMCs transfected with plasmid and siRNA targeting JunB were cultured in CM or CM with EGCG (20 μM) for 7 days. MAPK pathway-related protein expression in HASMCs of indicated groups was detected with western blotting. The quantification results are shown in Fig. S8. HASMCs, human aortic smooth muscle cells; α-SMA, alpha-smooth muscle actin; SM22α, smooth muscle 22 alpha; BMP2, bone morphogenetic protein-2; MSX2, msh homeobox 2; COL1A1, collagen type I alpha 1.

Osteogenic differentiation of VSMCs plays a pivotal role in vascular calcification, so we explored the effect of EGCG in this process. Immunofluorescence results revealed that the expression of VSMCs contractile phenotype markers alpha-smooth muscle actin and smooth muscle 22 alpha was significantly decreased, and osteoblastic differentiation-related markers alkaline phosphatase, runt-related transcription factor 2, and osteopontin, and matrix metalloproteinase 2/9 were up-regulated in calcified aortas. Meanwhile, EGCG reversed vascular calcification-associated protein change in calcified aortas (Fig. 1D; Fig. S1B, S2). Additionally, western blotting results showed that EGCG reversed the expression of vascular calcification-associated α-smooth muscle actin, alkaline phosphatase, and runt-related transcription factor 2 in HASMCs in a concentration-dependent manner (Fig. S3). In addition, quantitative real-time PCR results showed that co-treatment with EGCG (20 μM) decreased the mRNA levels of osteoblastic differentiation-related bone morphogenetic protein-2, msh homeobox 2, and collagen type I alpha 1 in HASMCs stimulated with calcifying medium for 2–14 days (Fig. 1E; Fig. S4). Overall, EGCG can inhibit VSMCs osteogenic differentiation both *in vivo* and *in vitro.*

The AP-1 transcription factor JunB is critical in the development of cardiovascular diseases such as atherosclerosis and acute aortic dissection.^5^ However, the role of JunB in vascular calcification remains unclear. In the present study, we found that JunB was significantly up-regulated in calcified mouse aortas and osteoblast-like HASMCs, whereas JunB was down-regulated after EGCG treatment (Fig. 1F, G). Furthermore, immunofluorescence staining demonstrated remarkably increased JunB nuclear translocation in calcified aortas. However, the translocation of JunB was significantly blocked by EGCG treatment (Fig. 1H). To further investigate whether JunB mediates the inhibitory effects of EGCG on vascular calcification, HASMCs were infected with plasmids and siRNAs targeting JunB in the presence of calcifying medium. The overexpression and knockdown of JunB in HASMCs were confirmed by western blotting analysis (Fig. S5). Alizarin Red S staining showed that JunB overexpression significantly exacerbated the process of vascular calcification and blocked the inhibitory effect of EGCG on HASMCs calcification. As a result, calcium salt deposition in HASMCs was further increased. However, the deposition of calcium salt in HASMCs was attenuated upon silencing JunB using siRNA, thereby augmenting the inhibitory effect of EGCG on calcification (Fig. 1I). These findings provide evidence that JunB exerts a pivotal role in driving the process of vascular calcification, while EGCG attenuates this pathological process by suppressing JunB expression.

The MAPK signaling pathway has been identified as a pivotal pathway implicated in vascular calcification, according to multiple studies. Western blotting results showed that the MAPK pathway was significantly activated in HASMCs in the presence of calcifying medium. However, EGCG treatment reduces the protein expression of the MAPK pathway, such as Ras, phosphorylation of the Ras-binding protein Raf1, p-MEK, and p-ERK in a concentration-dependent manner (Fig. 1J). Furthermore, the MAPK pathway was further activated by JunB overexpression. Of note, EGCG treatment decreased the protein expression of MAPK pathway markers, which was partly reversed by JunB overexpression (Fig. 1K). In contrast, the knockdown of JunB further reduced the expression of MAPK pathway-related proteins. Meanwhile, HASMCs with JunB knockdown significantly decreased the expression of p-Raf1, p-MEK, and p-ERK after EGCG treatment (Fig. 1L). These results indicate that EGCG inactivated the MAPK pathway by down-regulating the transcription factor JunB.

In summary, this study demonstrates that EGCG effectively ameliorates vascular calcification via modulation of the MAPK-JunB pathway. The down-regulation of JunB is essential for alleviating vascular calcification by EGCG. Enhancing EGCG levels through the consumption of green tea or administration of EGCG tablets holds promising potential as a therapeutic strategy to target JunB for preventing and treating vascular calcification.

## Ethics declaration

The animal experiments in this study were approved by the Tab of Animal Experimental Ethical Inspection of the Sichuan University (No. K2021015).

## Author contributions

TTL, FF, and EXW designed, conducted experiments, and drafted the manuscript. HMY, ZZ, and XXW analyzed and interpreted data. HCY and YS performed part of the experiments and supervised the project. GXW, WHH, and XHL provided general support and reviewed and edited the manuscript. All authors read and approved the final manuscript for publication.

## Conflict of interests

The authors declared no conflict of interests.

## Funding

This study was supported by the 10.13039/501100001809National Natural Science Foundation of China (No. 11932014, 12372315, 31971239), Sichuan Science and Technology Program (Sichuan, China) (No. 2022NSFSC0765, 2022ZYD0079, 2023YFS0297), and Science and Technology Innovation Project of JinFeng Laboratory, Chongqing, China (No. jfkyjf202203001).
