# Supplementary material for: EGCG alleviates vascular calcification via the MAPK/JunB signaling pathway
Source: Genes Dis. 2024 Feb 1;12(1):101237. doi: 10.1016/j.gendis.2024.101237 (PMC12053557; doi:10.1016/j.gendis.2024.101237)
Supplement: Multimedia component 1 [file mmc1.docx]

SUPPLEMENTARY MATERIALS FOR

**EGCG** **alleviates vascular calcification via the MAPK/JunB signaling pathway**

Tiantian Li^1^, Fei Fang^1^, Hongmei Yin^2^, Zhen Zhang^3^, Xiangxiu Wang^4,5^, Erxiang Wang^1^, Hongchi Yu^1^, Yang Shen^1^, Guixue Wang^4,5*^, Weihong He^6*^, Xiaoheng Liu^1,3*^

*^1^ Institute of Biomedical Engineering, West China School of Basic Medical Sciences & Forensic Medicine, Sichuan University, Chengdu 610041, China.*

*^2^ West China School of Pharmacy, Sichuan University, Chengdu 610041, China.*

*^3^Department of Cardiology, The Third People’s Hospital of Chengdu, Affiliated Hospital of Southwest Jiaotong University, Chengdu 610000, China.*

*^4^ Key Laboratory for Biorheological Science and Technology of Ministry of Education, State and Local Joint Engineering Laboratory for Vascular Implants, Bioengineering College of Chongqing University, Chongqing 400030, China.*

*^5^JinFeng Laboratory, Chongqing 401329, China.*

*^6^Department of Physiology, West China School of Basic Medical Sciences & Forensic Medicine, Sichuan University, Chengdu 610041, China.*

*Corresponding author:

Guixue Wang: wanggx@cqu.edu.cn

Weihong He: weihong.he@scu.edu.cn

Xiaoheng Liu: [liuxiaohg@scu.edu.cn](mailto:liuxiaohg@scu.edu.cn)

1. **Supplementary methods**

1.1 Animal experiments

Ten-week-old male C57BL/6J mice weighing 25-30g were purchased from Chengdu DOSSY Laboratory Animal Co. EGCG (MF: C_22_H_18_O_11_, MW: 458.37, purity: 98%) was purchased from Beijing Solarbio Technology Co., Ltd. (China). All mice were housed at the Laboratory Animal Center of Sichuan University (China). Following 1 week of acclimatization, the mice were randomized into three groups (12 per group) and treated as follows: group 1 (Ctrl group), 0.9% normal saline solution was given by gavage for 8 weeks; group 2 (VitD3 group), the medial arterial calcification model was induced by subcutaneous (s.c.) injection of 100 µL VitD3 (4×10^5^ U/kg bodyweight) once a day for three times as described; group 3 (VitD3 +EGCG group), the same dose of VitD3 was injected as in the VitD3 group and the EGCG (50 mg/d/kg body weight) was administered by gavage on the first day of injection for 8 weeks. The same standard chow and drinking water were provided to all groups of mice. Mice were anesthetized and euthanized at the end of the experiment. Blood and aorta samples were taken for later analysis.

1.2 Cell culture

Primary human aortic smooth muscle cells (HASMCs) were obtained from the ScienCell Research Laboratories (Sciencell, USA). HASMCs were cultured in a smooth muscle cell medium (SMCM, Sciencell, USA) at 37°C in a 95% humidified air, 5% CO2 incubator. HASMCs from 3 to 10 passages were used for the experiments.

1.3 HASMCs treatment

HASMCs were seeded in six-well plates or 60mm dishes and incubated for 2–21 days with growth medium (Ctrl); calcifying medium (CM, growth medium supplemented with 10 mM beta-glycerophosphate (β-GP, Sigma-Aldrich, USA), and 3 mM CaCl_2_); CM with EGCG (CM+EGCG) to induce calcification. EGCG at different concentrations (0, 0.1, 1, 5, 20, 30 µM) was used to treat HASMCs in CM for 7 days. The medium was changed every 2 days. In accordance with the experimental design, HASMCs in 6-well plates were analyzed for calcium deposition by Alizarin Red S staining on day 21. To determine the effect of EGCG on osteogenic differentiation of VSMCs, HASMCs were collected from 60 mm dishes to detect the protein and related gene mRNA expression by western blotting analysis and quantitative real-time PCR (qRT-PCR).

- 1. Plasmids and siRNAs Transfection

Small interfering RNAs (siRNAs) against human JunB (siJunB), scrambled siRNA (siNC), JunB overexpression plasmid (pEX-3 PGCMV/MCS/Neo), and vector plasmid (pEX1) were designed and synthesized by GenePharma (China). Lipofectamine reagent is a commonly used transfection reagent with high transfection efficiency and can be used to transfect plasmids or siRNAs into HASMCs.^1-3^ In this study, lipofectamine 8000 (Beyotime, China) was used to transfect specific plasmids and siRNAs (50 nM) into HASMCs according to the manufacturer's protocol. After 48 hours of transfection, the infection efficiency of the plasmids and siRNAs was confirmed by western blotting analysis. After 48 hours of transfection, HASMCs were switched to Ctrl, CM, CM with EGCG (20 μM) for calcification processing, followed by determination of protein expression and calcium deposition.

- 1. Alizarin Red S staining

For Alizarin Red S staining of HASMCs, the medium was removed and the cells were washed with PBS (Gibco, USA), and fixed in 95% ethanol for 10 minutes. HASMCs were washed 3 times with PBS and exposed to 1% Alizarin Red solution (pH 4.2, Solarbio, China) for 10 minutes at room temperature. To remove the excess dye, the HASMCs were rinsed three times with ultrapure water. An inverted microscope was used to take images. For Alizarin Red S staining of entire aortas, the entire aorta was fixed in 95% ethanol for 24 hours and then stained with 0.003% Alizarin Red S solution in 1% potassium hydroxide overnight with slow shaking. To remove excess dye from the tissue surface, the entire aorta was rinsed in 2% potassium hydroxide. To stain mouse aortic sections, 6 μm thick sections were deparaffinized, rehydrated, and exposed to 1% Alizarin Red S staining solution (pH 4.2, Solarbio, China) for 30 minutes. Wash with ultrapure water to remove the unbound dye. Positively stained areas showed a reddish color to indicate calcification.

- 1. von Kossa assay

The aortic sections were deparaffinized, rehydrated, and stained with a calcium salt stain (von Kossa method, Solarbio, China) according to the manufacturer's protocol. In brief, the sections were immersed in a 5% silver nitrate buffer and exposed to ultraviolet light for 30 minutes. Sections were rinsed in ultrapure water for 1 minute, followed by incubation in 5% sodium thiosulphate for 2 minutes. The nuclei of the cells were then stained with hematoxylin and eosin for 2 minutes. Wash with ultrapure water to remove the unbound dye. The slides were photographed by Olympus Slideview VS200 (Olympus, Japan).

1.7 Calcium content quantification

To quantify the calcium content in mouse aortas, the aorta was cut into small segments (~2 mm) and then incubated with 0.6N HCl at 4°C for 48 hours. The calcium content in the supernatants was determined with the calcium assay kit (Nanjing Jiancheng, Nanjing, China). After the assay of protein concentration using the BCA protein assay kit (Beyotime, China), the calcium content of the aortas was normalized to the total protein concentration.

1.8 Quantitative real-time PCR

TRIzol reagent (Life Technologies, USA) was used to extract total RNA from HASMCs. Synthesis of cDNAs was performed by reverse transcription of equal amounts (1000 ng) of RNA using a 5X Evo M-MLV RT reagent mix (Accurate Biology, China). qRT-PCR was performed using 2X Taq SYBR Green qPCR Mix (Innovagene, China) according to the manufacturer’s directions. Detection was performed using a CFX96 real-time PCR detection system (Bio-Rad, USA). The data were analyzed according to the 2- ^ΔΔCt^ method and the relative expression levels were normalized to the housekeeping gene GAPDH. The primer sequences for target genes are shown in Table S1.

1.9 Western blotting analysis

HASMCs and arterial tissues were lysed in a cold RIPA lysis Buffer (Solarbio, China) containing 1% PMSF, proteinase, and phosphatase inhibitors. The protein concentration in the supernatant was measured using an enhanced BCA protein assay kit (Beyotime, China) according to the manufacturer's directions. The lysed proteins were boiled in a loading buffer (Solarbio, China) at 100°C for 5 minutes. Equal amounts of protein (20-50 μg) were separated by 8–12% sodium dodecyl sulfate-polyacrylamide gel electrophoresis (SDS-PAGE) and transferred onto polyvinylidene fluoride (PVDF) membranes (Millipore, USA) at 4°C. After blocking with 5% skimmed milk for 2 hours at room temperature, membranes were incubated with primary antibodies at 4°C overnight.

Membranes were repeatedly washed with TBST, and subsequently incubated with HRP-conjugated anti-rabbit (diluted 1:10,000) secondary antibodies for 2 hours at room temperature. The membranes were rinsed three times with TBST for 10 minutes each, and the binding of the antibodies was detected using an ECL solution (4A Biotech, China). Quantification of protein blot intensities was performed using ImageJ. Gray-scale analysis was normalized to housekeeping protein (GAPDH) levels. The primary antibodies used in this study were anti-α-SMA (Cat #19245, Cell Signaling Technology), anti-SM22α (Cat #ab10135, Abcam), anti-ALP (Cat #ET1601-21, HUABIO), anti-Runx2 (Cat #D1L7F, Cell Signaling Technology), anti-OPN (Cat #41290, SAB), anti-JunB (Cat #49169, SAB), and goat anti-rabbit IgG secondary antibody (Cat #L3012, SAB).

1.10 Immunofluorescence staining

The aortic sections were deparaffinized, rehydrated, antigen retrieved, and then incubated with 5% goat serum for 1 hour at room temperature. Next, the sections were incubated with primary anti-α-SMA (1:200, Cat #19245, Cell Signaling Technology), anti-SM22α (1:200, Cat #ab10135, Abcam), anti-ALP (1:200, Cat #ET1601-21, HUABIO), anti-Runx2 (1:200, Cat #D1L7F, Cell Signaling Technology), anti-OPN (1:200, Cat #41290, SAB), and anti-JunB (1:200, Cat #49169, SAB) antibodies overnight at 4°C. After washing with PBS 3 times, the sections were incubated with secondary antibodies (Alexa Fluor 488, green) for 1 hour at room temperature, followed by incubation with DAPI (1:500, Solarbio, China) for 10 minutes at room temperature to stain the nucleus. The specimens were washed 3 times with PBS to remove the unbound dye. Finally, images were captured using a confocal laser-scanning microscope (LSM 710, Carl Zeiss, Germany).

1.11 Statistical analysis

For *in vivo* and *in vitro* experiments, the statistical analysis data are presented as mean ± standard error of the mean (SEM). GraphPad Prism (version 8.4.3) was used for statistical analysis. After assessing normality and equal variance, one-way ANOVA with post hoc Tukey’s honestly significant difference (HSD) analysis was used to compare multiple groups. All experiments were carried out independently at least three times and in triplicate each time. For all analyses, *P* values of < 0.05 were considered statistically significant. The statistical significance was represented as: **P* < 0.05, ***P* < 0.01, ^#^*P* < 0.05, ^##^*P* < 0.01, ns: not significantly different between indicated groups.

1. **Supplementary table**

**Table S1** The primers for human relative genes in this work.

| Gene name | Primers |
| --- | --- |
| Human BMP2 Forward (5’- 3’) | ACTACCAGAAACGAGTGGGAA |
| Human BMP2 Reverse (5’- 3’) | GCATCTGTTCTCGGAAAACCT |
| Human COL1A1 Forward (5’- 3’) | GTGCGATGACGTGATCTGTGA |
| Human COL1A1 Reverse (5’- 3’) | CGGTGGTTTCTTGGTCGGT |
| Human MSX2 Forward (5’- 3’) | CACCCTGAGGAAACACAAGAC |
| Human MSX2 Reverse (5’- 3’) | TGCACGCTCTGCAATGGAG |
| Human GAPDH Forward (5’- 3’) | ACAACTTTGGTATCGTGGAAGG |
| Human GAPDH Reverse (5’- 3’) | GCCATCACGCCACAGTTTC |

1. **Supplementary figures**


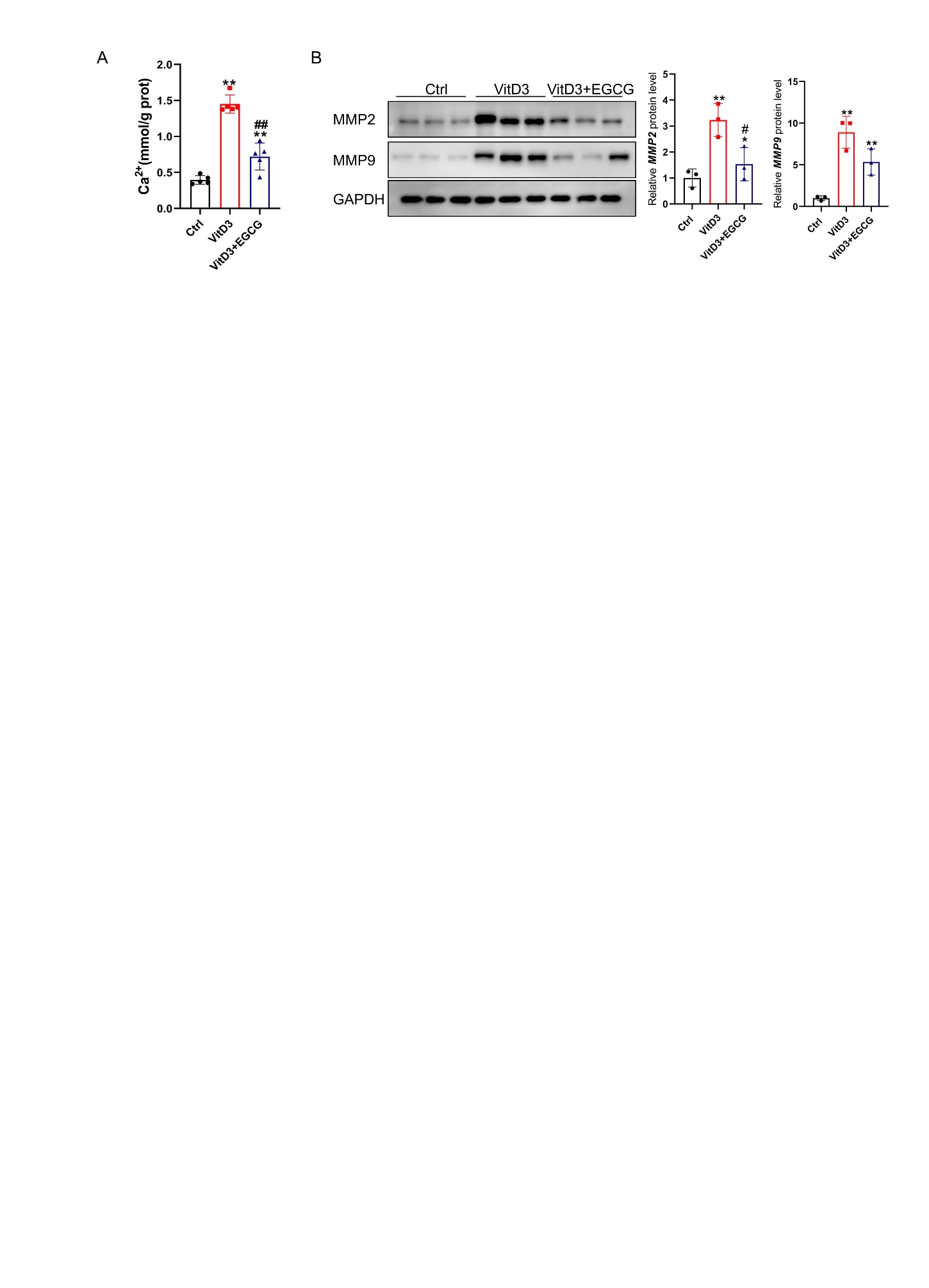


**Figure S1** EGCG reduced calcium deposition and the expression level of matrix metalloproteinases in VitD_3_-treated medial arterial calcification mice. **(A)** The quantitative analysis of calcium content in the mouse aortas (n = 5). **(B)** Western blotting analysis of matrix metalloproteinases MMP2 and MMP9 in the mouse aortas of Ctrl, VitD3, and EGCG treatment group (n = 3). The data are shown as mean ± SEM. **P* < 0.05, ***P* < 0.01 *vs.* Ctrl; ^#^*P* < 0.05, ^##^*P* < 0.01 *vs.* VitD3.


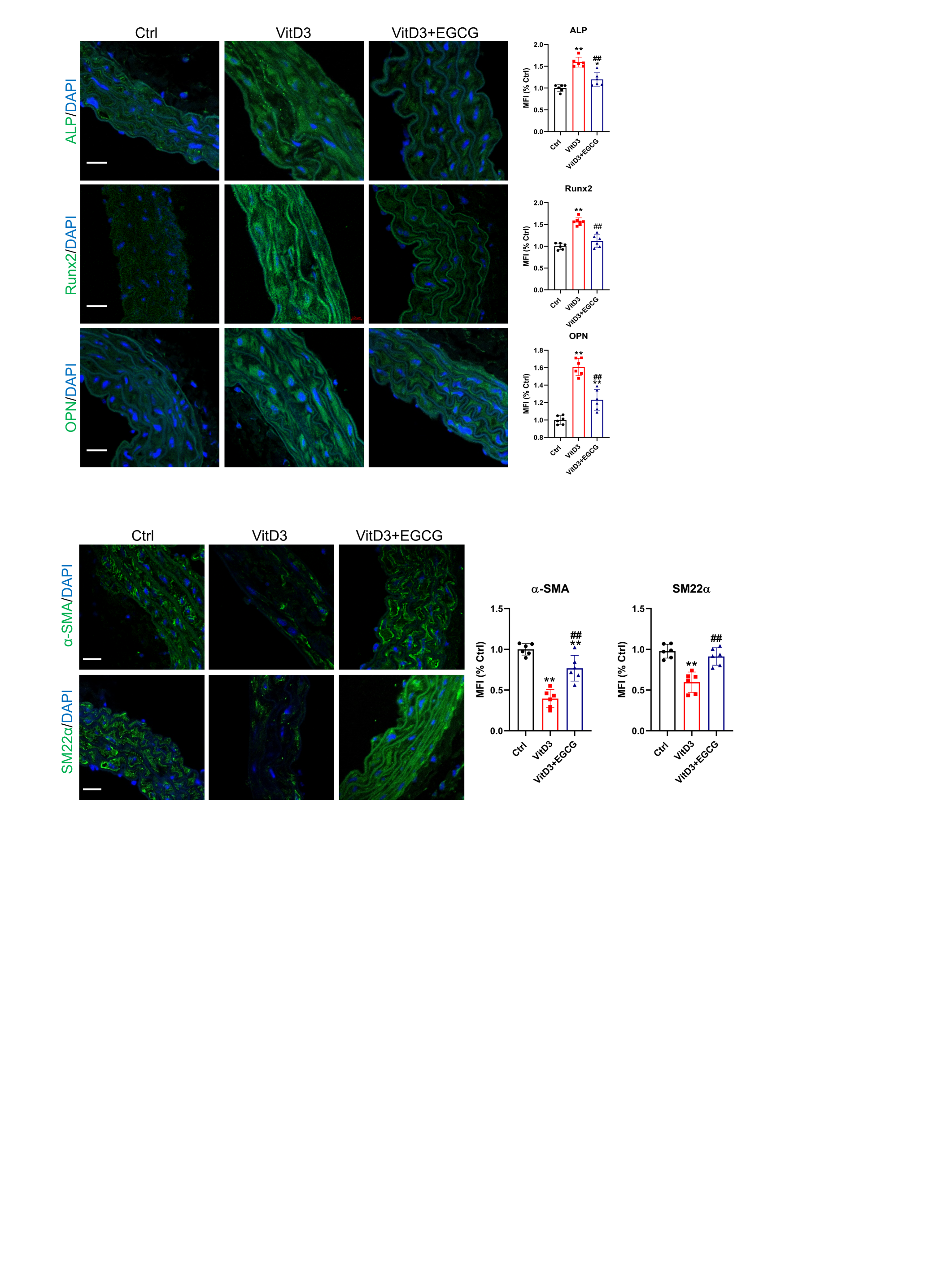


**Figure S2** EGCG inhibited VSMCs osteogenic differentiation in VitD_3_-treated mice aortas. The expression of VSMCs osteogenic markers ALP, Runx2, and OPN in mouse aortas was determined by immunofluorescent staining. ALP, Runx2, and OPN (green fluorescence); cell nuclei were stained with DAPI (blue fluorescence). Scale bar = 20 μm. The data are shown as mean ± SEM. **P* < 0.05, ***P* < 0.01 *vs.* Ctrl; ^#^*P* < 0.05, ^##^*P* < 0.01 *vs.* VitD3 (n = 6).


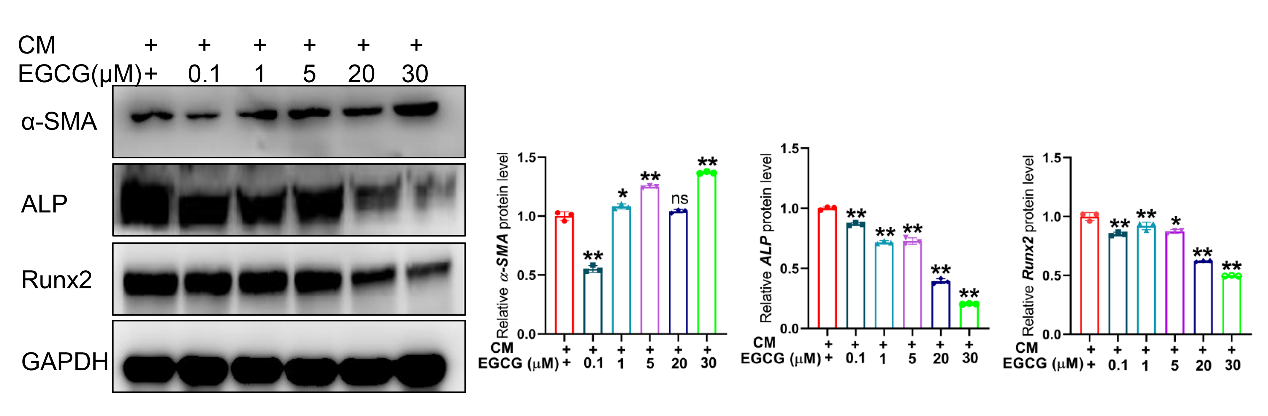


**Figure S3** EGCG inhibited the osteogenic differentiation of HASMCs *in vitro*. HASMCs were cultured in calcifying medium (CM) or CM with EGCG (0.1-30 μM) for 7 days. The expression of VSMCs vascular calcification-associated proteins in HASMCs was determined by western blotting. The data are shown as mean ± SEM. **P* < 0.05, ***P* < 0.01 *vs.* CM. ns: not significantly different between indicated groups (n = 3).

**
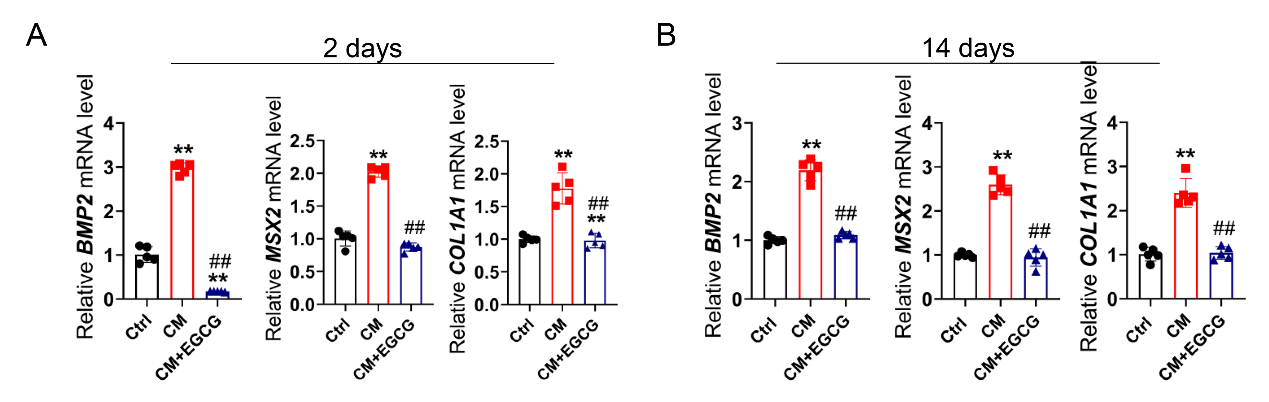
**

**Figure S4** EGCG inhibited the mRNA expression of VSMCs osteoblastic differentiation-related markers in HASMCs *in vitro*. **(A, B)** HASMCs were cultured in growth medium (Ctrl), calcifying medium (CM), or CM with EGCG (20 μM) for 2 and 14 days. The mRNA expression of VSMCs osteoblastic differentiation-related markers *BMP2*, *MSX2*, and *COL1A1* was determined by qRT-RCR. The data are shown as mean ± SEM. **P* < 0.05, ***P* < 0.01 *vs.* Ctrl; ^#^*P* < 0.05, ^##^*P* < 0.01 *vs.* CM (n = 5).


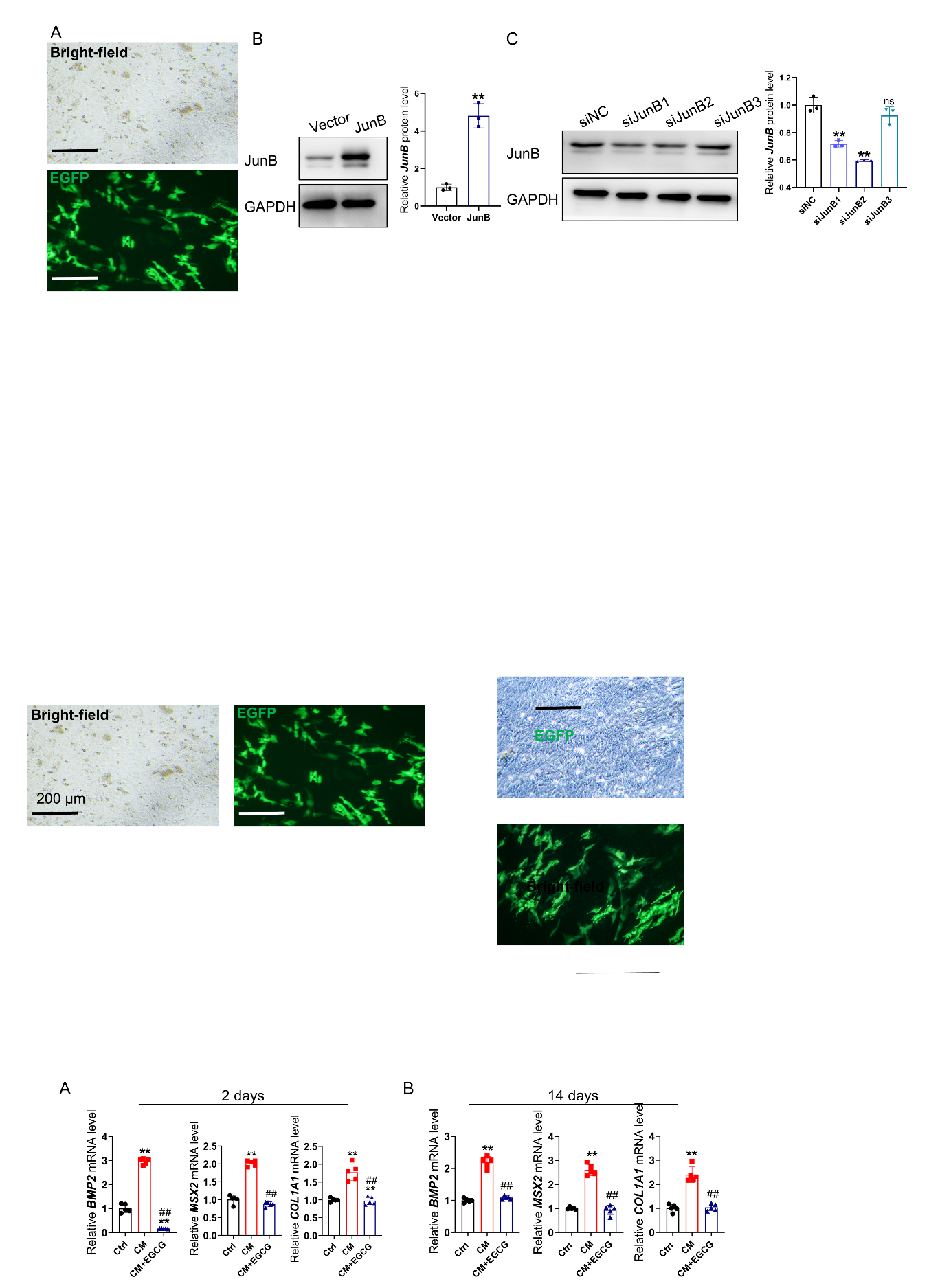


**Figure S5** To validate the success of JunB overexpression and knockdown in HASMCs. **(A)** Bright-field (BF) and enhanced GFP images of HASMCs transfected with vector plasmid for 2 days. (Sscale bar = 200 μm). **(B)** HASMCs transfected with vector plasmid and JunB overexpression plasmid were cultured in a growth medium for 2 days. Western blotting analysis of JunB-overexpression in HASMCs. **(C)** HASMCs transfected with three different siRNAs targeting JunB or negative control (siNC) were cultured in a growth medium for 2 days. The protein expression of JunB in HASMCs was analyzed by western blotting. The data are shown as mean ± SEM. **P* < 0.05, ***P* < 0.01 *vs.* indicated. ns: not significantly different between indicated groups (n = 3).


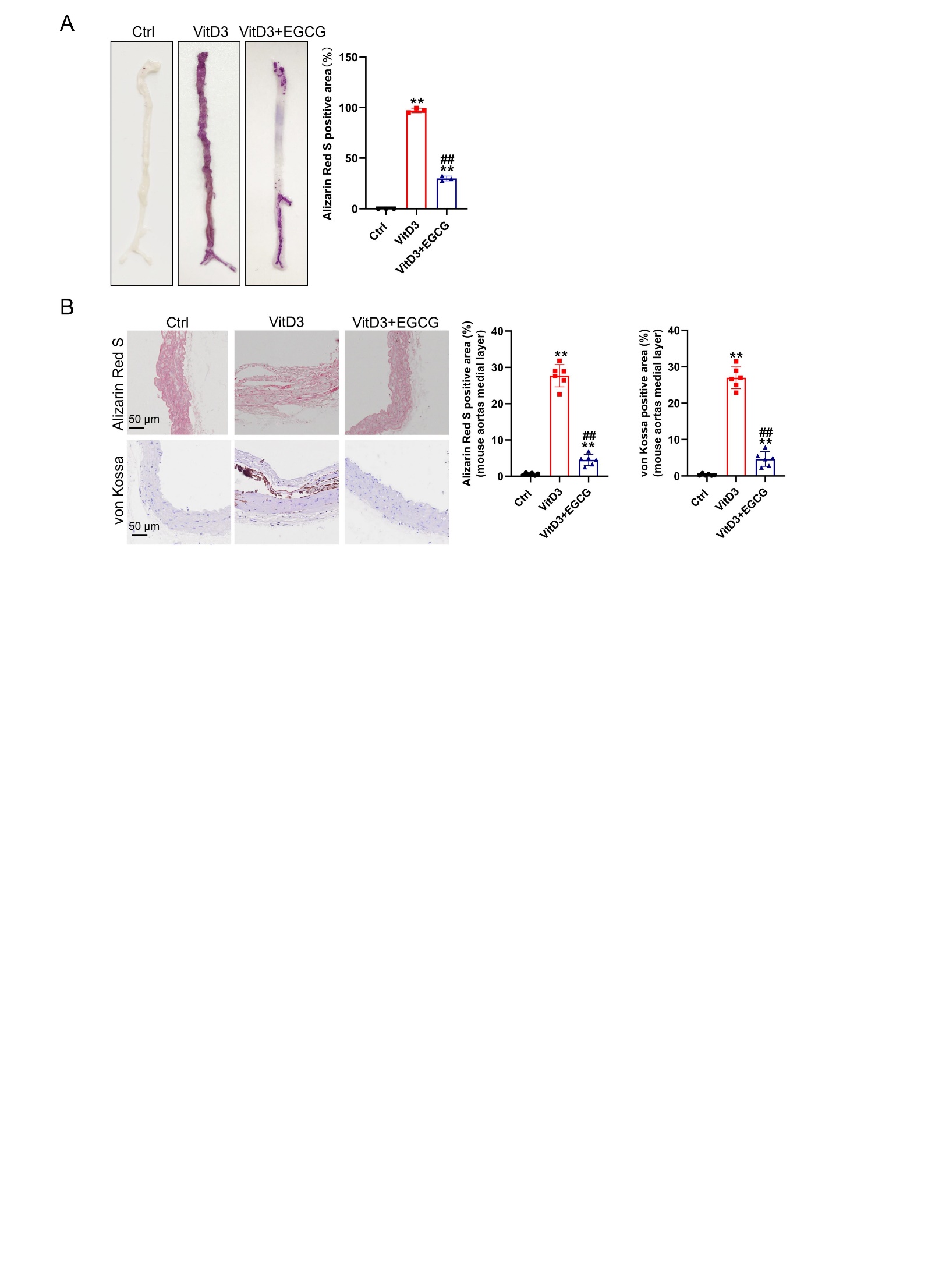


**Figure S6** The quantitative analysis of Alizarin Red S and von Kossa positive staining in Figure 1.

**(A)** Quantification of Alizarin Red S positive staining for Figure 1A (n = 3). **(B)** Quantification of Alizarin Red S and von Kossa positive staining for Figure 1B (n = 6). The data are shown as mean ± SEM. **P* < 0.05, ***P* < 0.01 *vs.* Ctrl; ^#^*P* < 0.05, ^##^*P* < 0.01 *vs.* VitD3.


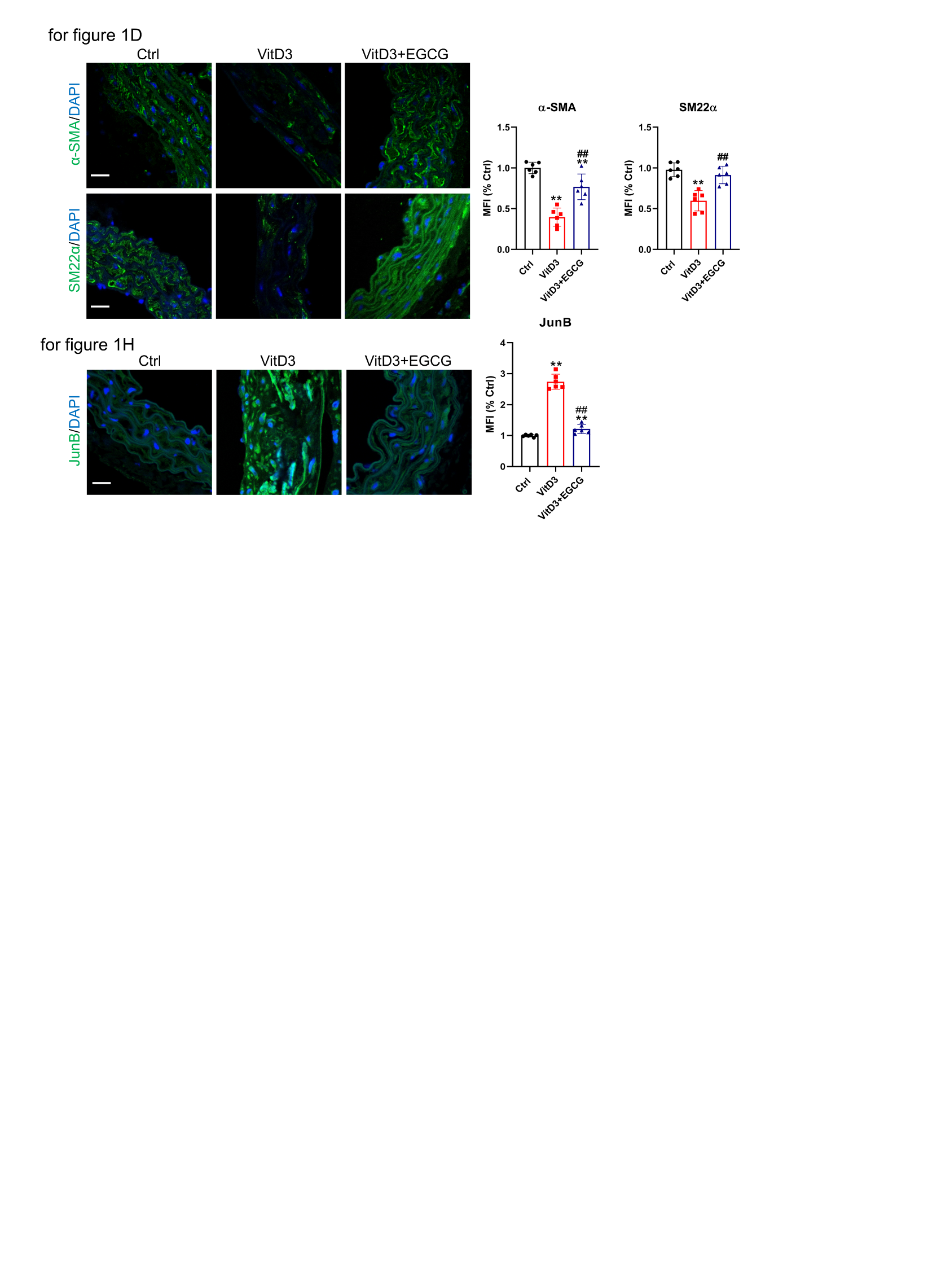


**Figure S7** The quantitative analysis of MFI in images for immunofluorescent staining in Figure 1. **(A)** Figure 1D. **(B)** Figure 1H. Scale bar = 20 μm. The data are shown as mean ± SEM. **P* < 0.05, ***P* < 0.01 *vs.* Ctrl; ^#^*P* < 0.05, ^##^*P* < 0.01 *vs.* VitD3 (n = 6).


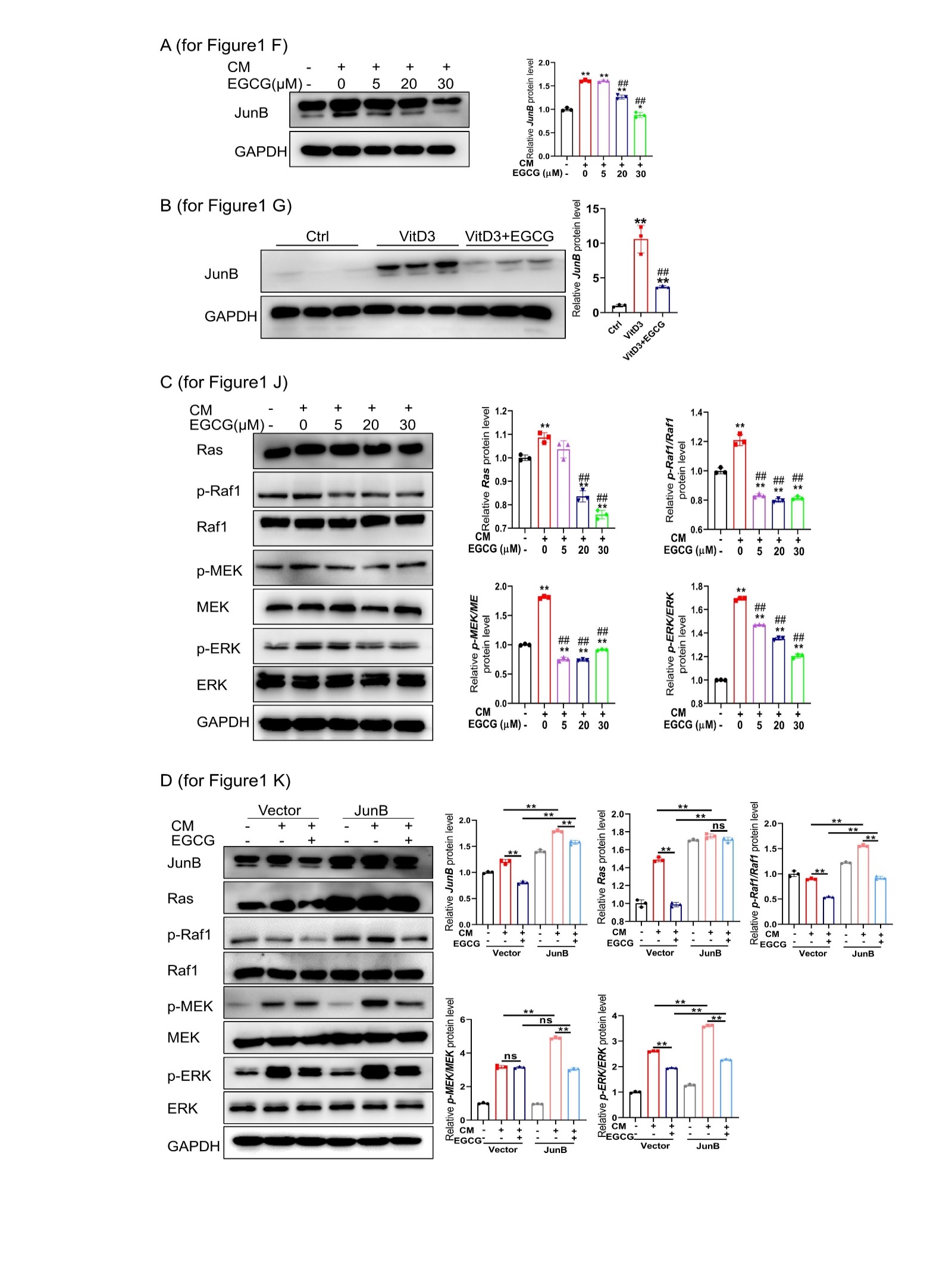


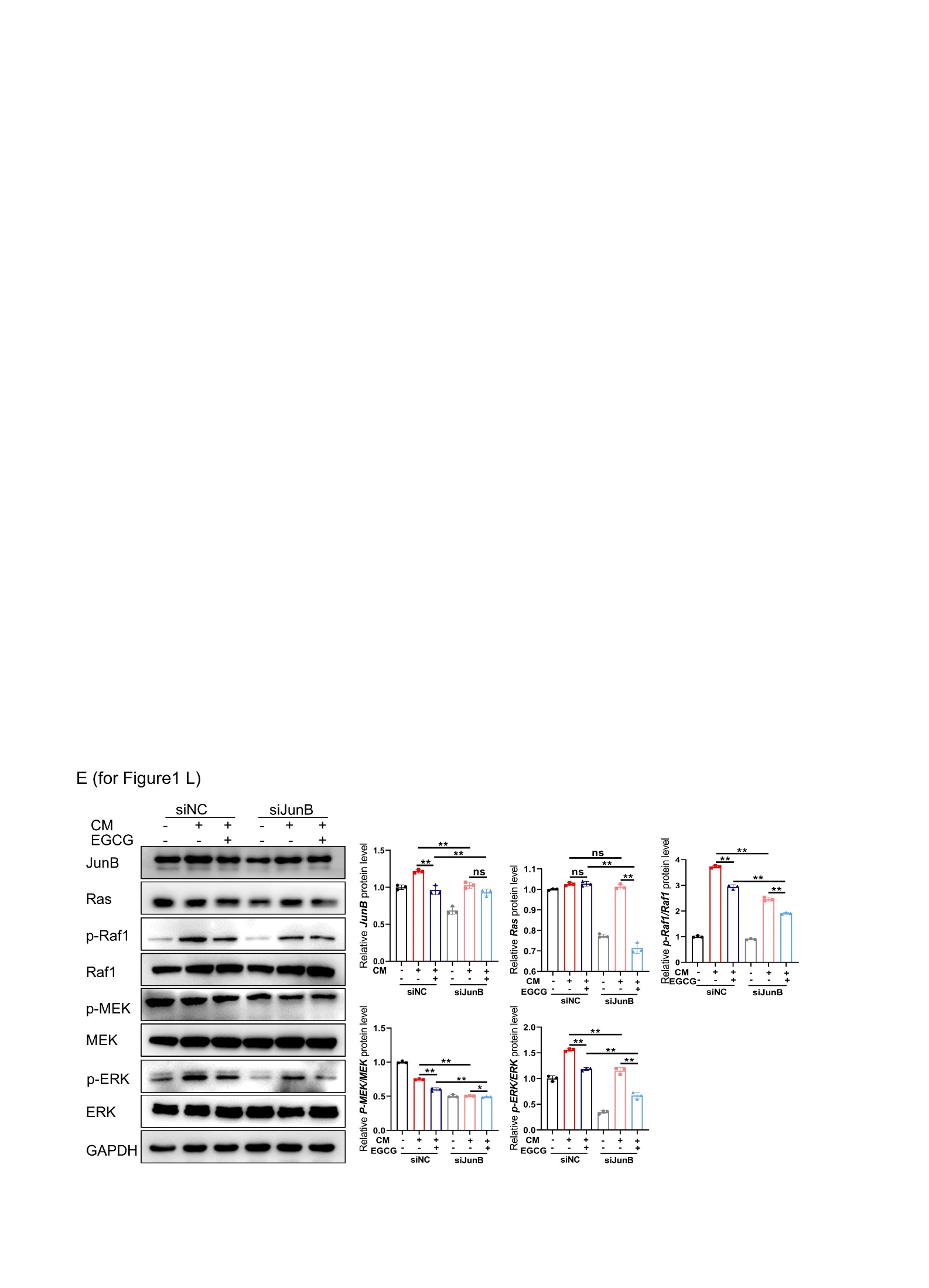


**Figure S8** The statistical analysis of band density for western blotting in Figure 1. All the images of western blotting were conducted band density analysis. The statistical results were used to make the dot-like graphs as shown in this figure. **(A)** Figure 1F. **(B)** Figure 1G. **(C)** Figure 1J. **(D)** Figure 1K. **(E)** Figure 1L. The data are shown as mean ± SEM. **P* < 0.05, ***P* < 0.01; ^#^*P* < 0.05, ^##^*P* < 0.01 *vs.* indicated. ns: not significantly different between indicated groups (n = 3).

**Reference**

1. Zhao XK, Zhu MM, Wang SN, et al. Transcription factor 21 accelerates vascular calcification in mice by activating the IL-6/STAT3 signaling pathway and the interplay between VSMCs and ECs. *Acta Pharmacol Sin.* 2023;44(8):1625-1636.

2. Wang Y, Zhang ZY, Chen XQ, Wang X, Cao H, Liu SW. Advanced glycation end products promote human aortic smooth muscle cell calcification in vitro via activating NF-κB and down-regulating IGF1R expression. *Acta Pharmacol Sin.* 2013;34(4):480-486.

3. Li X, Zheng T, Zhang Y, et al. Dickkopf-1 promotes vascular smooth muscle cell foam cell formation and atherosclerosis development through CYP4A11/SREBP2/ABCA1. *Faseb j.* 2023;37(8):e23048.
